# Maternal thyroid function in multiple pregnancies – a systematic review

**DOI:** 10.3389/fendo.2022.1044655

**Published:** 2023-01-17

**Authors:** Magdalena Zgliczynska, Magdalena Ostrowska, Iwona Szymusik, Michal Ciebiera, Katarzyna Kosinska-Kaczynska

**Affiliations:** ^1^ Department of Obstetrics, Perinatology and Neonatology, Centre of Postgraduate Medical Education, Warsaw, Poland; ^2^ Department of Endocrinology, Centre of Postgraduate Medical Education, Warsaw, Warsaw, Poland; ^3^ Second Department of Obstetrics and Gynecology, Centre of Postgraduate Medical Education, Warsaw, Poland

**Keywords:** thyroid, pregnancy, twin pregnancy, multiple pregnancy, human chorionic gonadotropin

## Abstract

**Background:**

The function of the thyroid gland during pregnancy undergoes physiological changes to ensure the proper amount of thyroid hormones for both the pregnant woman and the fetus. Multiple pregnancies (MP) are characterized by specific differences compared to single pregnancies, e.g., higher concentrations of human chorionic gonadotropin, which also affect thyroid function. The aim was to collect available knowledge on maternal thyroid function in MP.

**Methods:**

We have systematically searched three databases: the PubMed/MEDLINE, Scopus and the Cochrane Library. The last search was run on the 4th of August 2022. We included full-text original observational and experimental studies written in English. Case reports, editorials, letters, conference abstracts, reviews and meta-analyses were excluded. No time criterion was established. Studies were considered eligible if at least one maternal thyroid function test was performed and reported. Studies on MP with a co-existing mole were excluded. The risk of bias was assessed with the use of the AXIS tool. The qualitative synthesis of evidence was applied.

**Results:**

The search strategy resulted in the identification of 821 manuscripts. After removing duplicates, we screened the titles and abstracts of 552 articles, out of which 57 were selected for full-text analysis. Finally, 12 articles were included in the review. They were conducted in 6 different countries and published between the years 1997 and 2022. The number of examined women with MP ranged from 9 to 1 626.

**Discussion and conclusions:**

Thyroid function differs between women with MP and SP. Scarce data are available on the topic, but MPs are most likely characterized by higher HCG levels, which influences thyroid-stimulating hormone and free thyroid hormone levels. These differences are mainly expressed in the 1st trimester of pregnancy. Separate population-based reference ranges are needed to correctly diagnose thyroid diseases in MP and to avoid unnecessary treatment. Further research is needed to fill the knowledge gaps.

## 1 Introduction

The thyroid is an endocrine gland whose main function is the secretion of thyroid hormones, mainly triiodothyronine (T3) and thyroxine (T4). The above-mentioned thyroid hormones (TH) exert a wide range of effects in the human body, primarily acting as metabolism and growth regulators in almost all tissues. Their secretion is regulated by the thyroid-stimulating hormone (TSH), which is secreted by the anterior pituitary gland, whereas TSH secretion is further controlled by thyrotropin-releasing hormone (TRH) produced by the hypothalamus and by TH *via* negative feedback (1).

The function of the thyroid gland during pregnancy undergoes significant physiological changes to ensure the proper amount of TH for both the pregnant woman and the developing fetus (2). Importantly, TH takes part in the development of the fetal brain by regulating the migration, proliferation, differentiation and myelination of neuronal cells and the synapse formation (3). An extreme state of TH deficit caused by severe iodine deficiency during the prenatal period leads to cretinism, a symptom complex which is primarily manifested as severe intellectual disability (4). Preserving maternal euthyroidism is especially important in the first half of pregnancy (5). The fetal thyroid becomes fully functional around the 20^th^ week of gestation, so the fetus remains completely dependent on maternal supply until that time (5, 6). Under normal conditions, several mechanisms contribute to the proper distribution of TH. Primarily, human chorionic gonadotropin (HCG) produced by the placental trophoblast, structurally similar to TSH by containing an identical alpha subunit (α-HCG) and a specific beta subunit (ß-HCG), acts as a weak agonist on the TSH receptor (TSHR) and becomes the main thyrotropic factor during the first trimester (7). It results in the so-called mirror image – a gradual decrease in TSH level in response to an increase in HCG concentrations, especially expressed in the 1^st^ trimester (8). Conversely, high levels of estrogens contribute to an increase in thyroxine-binding globulin (TBG) levels, which results in a 50% rise in the total fraction of TH (9). Another regulatory mechanism is related to the activity of placental deiodinases, especially placental deiodinase type 3. This enzyme catalyzes the conversion of T4 and T3 to inactive metabolites: reverse triiodothyronine (rT3) and diiodothyronine (T2), which facilitates the regulation of fetal exposure to maternal TH (10). The above-described adjustments, along with an increased demand for iodine, might exacerbate maternal thyroid dysfunctions making them latent until that moment.

Various data are available on the incidence of intragestational thyroid diseases, especially as regards subclinical hypothyroidism, which is partially related to the adoption of different reference ranges (11). Notably, nowadays the topic seems so debatable that essential differences in care are pronounced not only between scientific societies but also between different clinical centers and individual healthcare providers (12). Consequently, it is increasingly postulated to use population-specific reference ranges for TSH and TH levels in pregnancy. It seems particularly important, since unnecessary treatment, especially thyrostatic, exposes the mother and the fetus to potential side effects of the therapy (13). Numerous factors may impact the reference values in different populations, primarily: body mass index, iodine supply, ethnicity, the presence of anti-thyroid antibodies. Numerous other factors may play a role, e.g., the presence of focal lesions in the thyroid gland (14, 15). Therefore, specific reference values are currently being developed by various scientific societies but they are still insufficiently widespread (14, 16, 17). Nevertheless, such studies most often excluded the population of patients with multiple pregnancies (MP) from further calculations since they were characterized by specific differences (18). In the context of thyroid function, these mostly include higher concentrations of HCG and estrogens (19). Moreover, given the increased metabolic requirements, women with MP may constitute a risk group as regards various nutritional deficiencies (20). It was also demonstrated that they were more likely to suffer from nausea and vomiting of pregnancy (NVP). However, the relationship between NVP and the deficiency of iodine, the most important micronutrient in the context of proper thyroid function, has not been fully elucidated yet (21–24). Therefore, it should be considered that disturbances in thyroid function in this particular group of pregnant women may occur more frequently which was demonstrated by Chen et al. on a large group of patients with twin pregnancies (TP). The study showed that TPs were associated with a higher risk of overt hyperthyroidism and isolated hypothyroxinemia in early pregnancy, but the risk of subclinical hypothyroidism was lower than in singleton pregnancy (SP). Later in pregnancy, the authors associated TP with a higher risk of subclinical hypothyroidism, isolated hypothyroxinemia and subclinical hyperthyroidism (25).

Considering the above, the aim of this systematic review is to gather currently available knowledge on maternal thyroid function in MP.

## 2 Methods

This systematic review was developed in correspondence with the updated Preferred Reporting Items for Systematic Reviews and Meta-Analyses Statement (PRISMA) dated from 2020 (26, 27). We have systematically searched three databases: the PubMed/MEDLINE, Scopus and the Cochrane Library. Articles found in the databases were considered regardless of the publication date. The last search was initiated on the 4^th^ of August 2022. The detailed search design is presented in **Table 1**.

**Table 1:** Databases used and corresponding search lines.

**PubMed/** **MEDLINE**	315	(“Pregnancy, Twin”[Mesh] OR “Pregnancy, Multiple”[Mesh] OR (“twin pregnancy”) OR (“twin pregnancies”) OR (“twin gestation”) OR (“twin gestations”) OR (“triple pregnancy”) OR (“triple pregnancies”) OR (“triple gestation”) OR (“triple gestations”) OR (“multiple pregnancy”) OR (“multiple pregnancies”) OR (“multiple gestation”) OR (“multiple gestations”) OR (“multifetal”) OR (“dizygotic”) OR (“monozygotic”)) AND (“Thyroid Gland”[Mesh] OR thyroid*)
**Scopus**	481	TITLE-ABS-KEY (((“twin pregnancy”) OR (“twin pregnancies”) OR (“twin gestation”) OR (“twin gestations”) OR (“triple pregnancy”) OR (“triple pregnancies”) OR (“triple gestation”) OR (“triple gestations”) OR (“multiple pregnancy”) OR (“multiple pregnancies”) OR (“multiple gestation”) OR (“multiple gestations”) OR (“multifetal”) OR (“dizygotic”) OR (“monozygotic”)) AND (thyroid*))
**Cochrane**	25	#1 “Pregnancy, Twin”[Mesh] #2 “Pregnancy, Multiple”[Mesh] #3 (“twin pregnancy”) OR (“twin pregnancies”) OR (“twin gestation”) OR (“twin gestations”) OR (“triple pregnancy”) OR (“triple pregnancies”) OR (“triple gestation”) OR (“triple gestations”) OR (“multiple pregnancy”) OR (“multiple pregnancies”) OR (“multiple gestation”) OR (“multiple gestations”) OR (“multifetal”) OR (“dizygotic”) OR (“monozygotic”) #4 “Thyroid Gland”[Mesh] #5 thyroid* (#1 OR #2 OR #3) AND (#4 OR #5) and Trials

the asterisk (*) represents any group of characters, including no character.

The search retrieved 821 manuscripts. We used the automatic duplicate identifier function built in EndNote X9 (Clarivate Analytics, London, UK). As a consequence, 222 duplicates were removed, while another 47 items were manually deleted. The remaining articles (599) were independently screened by two study authors. The next step included eligibility assessment that was performed by two other authors. We included full-text original observational and experimental studies written in English, and excluded case reports, editorials, letters to the editor, conference abstracts, as well as reviews and meta-analyses. Studies were considered eligible if at least one maternal thyroid function test was performed (for example TSH, FT3, FT4) and reported. MPs with a co-existing mole were excluded. Any disagreement which occurred between study authors was resolved through discussion in the presence of all other authors.

**Figure 1:**
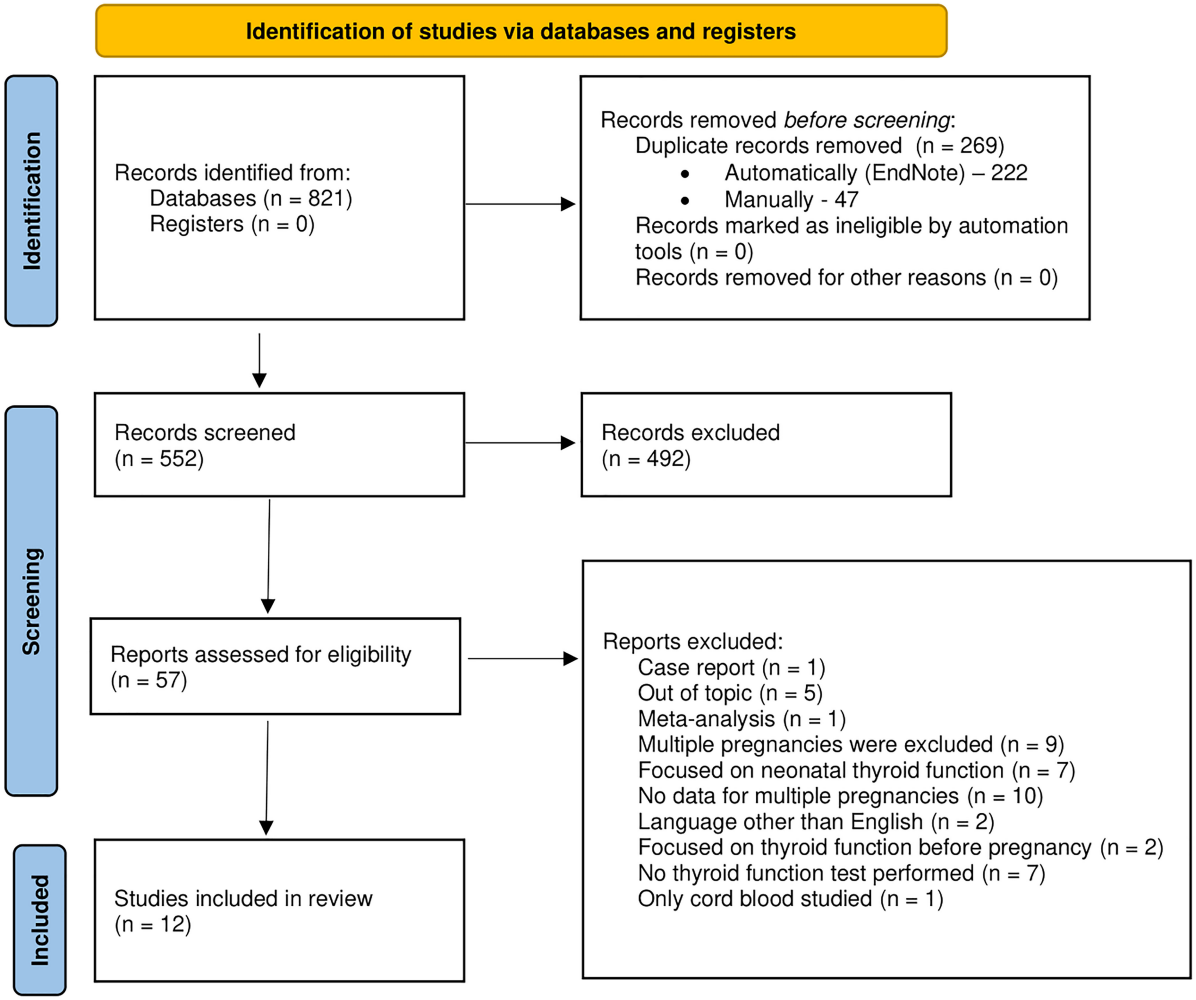
The PRISMA 2020 flow diagram for new systematic reviews which included searches of databases and registers only.

The entire selection process is presented in **Figure 1** using the PRISMA 2020 flow diagram.

Using a custom-built data extraction form we sought the following information: the authors, year and country of origin, type of study, the main objective, population characteristics, obstetrical data, diagnostic tools and methods used, study timepoints and the results. Two study authors extracted the above-mentioned data from the selected full-text articles, while the third author double-checked their accuracy. No quantitative analyses were performed due to the significant diversity of the surveyed populations and research methodologies.

We attempted to contact the authors of several studies in order to obtain detailed information on their projects: Huang et al., Zhang et al., Liu et al., Rosner et al. and Chen et al. (25, 28–31). We have received answers from: Zhang et al. and Rosner et al. (29, 31).

The risk of bias was assessed with the use of the AXIS tool (32).

## 3 Results

### 3.1 General characteristics of identified studies

The implemented search strategy facilitated the identification of 12 eligible articles (25, 30, 31, 33–41). They were published between 1997 and 2022. At least 4330 women with TP and 48 women with MP were studied. As regards the selected studies: 3 were conducted in China (25, 30, 40), 2 in the United Kingdom (35, 37), 2 in the United States of America (31, 36), 2 in the Czech Republic (39, 41), 2 in Japan (34, 38) and 1 in Belgium (33). The number of examined women with MP ranged from 9 to 1 626 in individual studies. All the studies included TSH measurement results (25, 30, 31, 33–41), 11 out of 12 – FT4 results (25, 30, 31, 33–35, 37–41), and 2 out of 12 – FT3 results (34, 38). Half of the studies concurrently examined HCG and/or its subunits (25, 33–35, 37, 38). Only 2 studies included patients pregnant with more than two fetuses, with the maximum of 8 fetuses (34, 35). 4 studies aimed at establishing reference ranges for TP (36, 37, 39, 40).

The basic characteristics of the retrieved studies are presented in **Table 2**. A report on the methodology of laboratory examinations implemented in each study can be found in the **Supplementary Materials** (**Supplementary Material 1**).

**Table 2:** Basic characteristics of the retrieved studies.

Authors, year and country of publication	Main aim of the study	Population of pregnant women	Data on thyroid function	Parameters	Time-points
**Grün, Meuris et al.** 1997 Belgium	To examine the thyrotropic role of HCG in early TP	17 SP **13 TP** All after IVF	No history of thyroid disease, no ATA	HCG, α-HCG, β-HCG, TSH, FT4, ATA	GWs: 6, 8, 9, 10, 11, 15, 19, 22, 32
**Sakaguchi, Yoshimura et al.** 1998 Japan	To evaluate thyroid function and the thyrotropic action of HCG in MP	27 SP (age 23-38) **9 MP** **(6 TP, 3 TrP)** (age 26-37)	No history of thyroid disease	HCG, α-HCG, β-HCG, TSH, FT3, FT4, TSA	Trimesters: 1^st^, 2^nd^, 3^rd^
**Ogueh, Hawkins et al.** 2000 the United Kingdom	To examine maternal TSH, FT4 and HCG in MP before and after embryo reduction	*group 1* (12 SP, **12 TP** after IVF-ET) *group 2* **(39 MP (3-8 fetuses))** undergoing fetal reduction to TP)	No data	TSH, FT4, HCG	GWs: 10–12 (before fetal reduction), 4 weeks after, 8 weeks after
**Dashe, Casey** **et al.** 2005 the United States of America	To estimate the reference range for TSH in SP and TP	13 599 SP (89% aged 18-35) **132 TP** (90% aged 18-35)	No data	TSH	42% by 12 GWs, 69% by 20 GWs, 95% by 37 GWs
**Ashoor, Muto** **et al.** 2013 the United Kingdom	To establish reference ranges of maternal serum TSH and FT4 at 11–13 GWs of TP	**235 TP** (**177 DC**; (median age 33.7), **58 MC** with normal outcomes (median age 33.6), **19 MC** complicated by severe TTTS (median age 32.6)	No history of overt thyroid disease; 84.3% negative TPOAb and TgAb	β-HCG, TSH, FT4, TPOAb, TgAb	GWs: 11-13 DC & MC median 13.0 TTTS median 12.5
**Hanaoka, Arata et al.** 2015 Japan	To study the relationship between maternal serum HCG and thyroid function in TTTS	**131 TP** All with TTTS = MC	No data	HCG, TSH, FT3, FT4	GWs: 16-26 (before operation) 2 weeks after, 4 weeks after
**Rosner, Fox et al.** 2017 the United States of America	To determine if the treatment of overt hypothyroidism in TP reduced the adverse outcomes	**612 TP** Hypothyroid (n = 85; mean age 35.3) Healthy n = 527 (mean age 34.0)	13.9% of overt hypothyroidism at treatment initiation, all treated until TSH reached <2.5 IU/ml	TSH, FT4	At the beginning of prenatal care (not specified)
**Šálek, Dhaifalah et al.** 2018 the Czech Republic	To establish maternal TSH reference ranges for 1^st^ trimester screening from 11 + 0 to 13 + 6 GWs	10 592 SP (median age 29) **201 TP** (median age 31)	No history of thyroid disease Adequate iodine supply assumed (iodized salt in the Czech Republic since 1947)	TSH, FT4, TPOAb	SP median 12 GWs + 4 days TP median 12 GWs + 5 days
**Šálek, Dhaifalah et al.** 2019 the Czech Republic	To determine the prevalence of maternal hypothyroidism at 11-14 GWs and to compare the rates for SP and TP	4 965 SP (median age 30) **109 TP** (median age 31)		TSH, FT4, TPOAb	SP & TP median 12 GWs + 5 days
**Jiang, Sun et al.** 2019 China	To establish reference ranges for thyroid-related indicators in early TP and to compare with SP	480 SP (median age 30) **160 TP** (median age 31) • n = 21 at 4-6 GWs • n = 139 at 7-12 GWs	No history of thyroid disease or thyroid-interfering medications, IVF, TPOAb positivity, adequate iodine supply assumed basing on (42, 43)	TSH, FT4, TPOAb	SP & TP median 9 GWs
**Chen, Yang et al.** 2021 China	To evaluate thyroid function in TP and compare with SP	46 834 SP (median age 30) **1 208 TP** (median age 31)	No history of thyroid disease, TPOAb positivity, SP - 10.3% TP - 10.8%	HCG, TSH, FT4, TPOAb	Early GWs: 8-14 Late GWs: 28-35
**Liu, Su et al.** 2022 China	To study the association of TH and birth weight in TP	**1 626 TP** (81.1% <35 years) 72.4% DC 27.6% MC	No history of thyroid disease or taking thyroid-interfering medications, TTTS or ART	TSH, FT4, TPOAb	64.1% in the 1^st^ trimester 32.8% in the 2^nd^ trimester 3.1% in the 3^rd^ trimester

ART, assisted reproductive techniques; ATA, anti-thyroid antibodies; DC, dichorionic; GW, gestational week; HCG, human chorionic gonadotropin; IVF-ET, in vitro fertilization and embryo transfer; FT3, free triiodothyronine; FT4, free thyroxine; MC, monochorionic; MP, multiple pregnancies; SP, single pregnancies; TFT, thyroid function test; TgAb, anti-thyroglobulin antibodies; TH, thyroid hormones; TP, twin pregnancies; TPOAb, anti-thyroid peroxidase antibodies; TSA, thyroid stimulating activity; TSH, thyroid stimulating hormone; TTTS, twin-to-twin transfusion syndrome.

**Table 3** presents data on the levels on TSH and TH in MP.

**Table 3:** Thyroid function test results in multiple pregnancies.

Authors and year of publication	Adopted criteria/reference ranges	TSH	FT4 and FT3	Thyroid diseases
**Grün, Meuris et al.** 1997	TSH: 0.2-4.0 mU/l FT4: 10-26 pmol/l	• TSH decreased progressively until 10-11 GWs in SP and TP • TSH reduction appeared deeper and more prolonged in TP, however, no statistical difference was confirmed at each time point • 6/10 TP and 3/14 SP reached TSH ≦̸0.2 mU/l	• An elevation in FT4 appeared ↑ in TP, however, no statistical difference was confirmed at each time point • There was a rise in FT4 between 10-18 GWs in TP, while in SP, FT4 slightly decreased during pregnancy, however, no statistical difference was found at each time point • Pooled FT4 was ↑ between 8-15 GWs in TP than in SP • Although FT4 remained normal in SP, it was transiently supranormal in 4/13 TP	Not studied
**Sakaguchi, Yoshimura et al.** 1998	TSH: 0.4-4.7 mU/l FT4: 1.0-1.8 ng/dl FT3: 2.5-4.3 pg/ml	• In both SP and MP serum TSH levels were lowest in the 1^st^ trimester • No difference was found in TSH levels between SP and MP in each trimester	• The mean FT3 and FT4 concentrations in MP did not differ from SP in each trimester	Not studied
**Ogueh, Hawkins et al.** 2000	No data	• There was no difference in TSH between SP and TP (at 10–12, 14–16 and 18–20 GWs), • Before fetal reduction, in MP TSH was ↓ compared to SP, but not to TP • After reduction to TP, TSH in MP was no different to TP values by 8 weeks	• There was no difference in FT4 between SP and TP (at 10–12, 14–16 and 18–20 GWs) • Before fetal reduction, FT4 was ↑ in MP than in SP or TP • After reduction to TP, FT4 decreased progressively in MP, but remained ↑ than in TP at 4 weeks after reduction	Not studied
**Dashe, Casey** **et al.** 2005	TSH: ± 2SD for the mean for GW on nomogram *For details see* **Figure 1** *in the original manuscript*	• The decrease in TSH during the 1^st^ trimester was ↑ in TP than in SP • In the 1^st^ half of pregnancy, the threshold for identifying TSH elevation was approximately 0.4 mU/L ↓ in TP than in SP • For 1^st^ trimester SP, the approximate upper limit of normal TSH was 4.0 MoM, and for TPs – 3.5 MoM; thereafter, the approximate upper limit was 2.5 MoM for SP and TP	Not studied	Not studied
**Ashoor, Muto** **et al.** 2013	Population-based: TSH and FT4 (2.5-97.5^th^ pc of the healthy population: no thyroid disease, no DM, fetal abnormalities, PE, SGA, LBW), and depending on ethnicity, BMI and age *For details see* **Table 2** *and* **Table 3** *in the original manuscript*	• TSH MoM was ↓ in the ATA-negative TP with normal outcomes than in SP • TSH was no different between MC and DC • In the TTTS ATA-negative group, TSH was no different compared to normal TP *For details see* **Table 2** *and* **Table 3** *in the original manuscript*	• FT4 MoM was not different in the normal TP ATA-negative group than in SP • FT4 was not different between MC and DC • FT4 was not different in the TTTS ATA-negative group compared to normal TP	Not studied
**Hanaoka, Arata et al.** 2015	Hyperthyroidism: increased FT3 or FT4 levels with decreased or suppressed TSH	• TSH remained suppressed during 4 weeks after laser therapy	• When laser therapy was effective, FT3 and FT4 decreased progressively with HCG	Not studied
**Rosner, Fox et al.** 2017	Overt hypothyroidism – TSH >2.5 IU/mL and decreased FT4 based on the reference value provided *via* laboratory testing	The average level of TSH was 1.22 mIU/L	The average level of FT4 was 1.21 ng/dL	• 14% of TP had overt hypothyroidism at entry to healthcare • TP with overt hypothyroidism were more likely to have had IVF and pregestational diabetes • There was no difference in overt hypothyroidism rates between MC and DC
**Šálek, Dhaifalah et al.** 2018	**Population-based**: for TSH and FT4 (2.5-97.5^th^ pc of the healthy part of the Caucasian population: no thyroid disease or TPOAb positivity) **TSH** [mU/L] SP 11-13+6 GWs: 0.16-3.43 **TP** 11-13+6 GWs: 0.02-2.95 **FT4** [pmol/L] SP 11-13+6 GWs: 11.8-18.4 **TP** 11-13+6 GWs: 12.2-23.2	• In TP TSH during 1^st^ trimester screening was ↓ than that in SP *For details see* **Figure 2** *in original manuscript*	No details provided	Not studied
**Šálek, Dhaifalah et al.** 2019		No details provided	• FT4 was similar in TP and SP	• TP was associated with similar hypothyroidism prevalence as SP (6.4% vs 5.3%). However, all cases with TP were subclinical, while 92.4% of SP cases were subclinical and 7.6% were overt
**Jiang, Sun et al.** 2019	**TP Population-based**: for TSH and FT4 (2.5-97.5^th^ pc of the healthy population: no thyroid disease, thyroid-interfering drugs or TPOAb positivity) *4-12 GWs* **TSH**: 0.01–3.35 mIU/L **FT4:** 12.45–23.34 pmol/L *4-6 GWs* **TSH**: 0.06–3.25 mIU/L **FT4:** 13.28–19.86 pmol/L *7-12 GWs* **TSH**: 0.01–3.28 mIU/L **FT4:** 12.31–23.61 pmol/L	• In TP, TSH during 7-12 GWs was **↓** than that during 4-6 GWs • Between 4-12 GWs, TSH was **↓** in TP than in SP • Between 4-6 GWs, TSH was **↓** in TP than in SP • Between 7-12 GWs, TSH was **↓** in TP than in SP	• In TP, no difference was observed in FT4 levels between 7-12 GWs vs 4-6 GWs • In TP, an increasing trend was found in FT4 between 4-6 GWs; however, it was not statistically different from SP • Between 7-12 GWs, FT4 was ↑ in TP than in SP	Not studied
**Chen, Yang et al.** 2021	**SP Population-based**: for TSH and FT4 (2.5-97.5^th^ pc of the healthy population: **no TPs**, IVF, thyroid disease, thyroid-interfering drugs or TPOAb positivity) **TSH** early: 0.03-3.60 mU/L late: 0.39-3.69 mU/L **FT4** early: 11.7-19.8 pmol/L late: 9.1-14.4 pmol/L	• During early pregnancy TP was associated with ↓ TSH than SP • During late pregnancy TP was associated with ↑ TSH than SP	• During early pregnancy TP was associated with ↑ FT4 than in SP • During late pregnancy TP was associated with comparable FT4	**At GWs 8-14** TP was associated with: • ↑ risk of overt hyperthyroidism, subclinical hyperthyroidism and isolated hypothyroxinemia, • ↓ risk of subclinical hypothyroidism, • The rate of subclinical hypothyroidism was 0.7%, overt hypothyroidism 1.8%, isolated hypothyroxinemia 4.7%, subclinical hyperthyroidism 7.2%, overt hyperthyroidism 8.5%, **At GWs 28-35** TP was associated with: • ↑ risk of subclinical hypothyroidism, isolated hypothyroxinemia and subclinical hyperthyroidism • The rate of subclinical hypothyroidism was 8.2%, overt hypothyroidism 1.3%, isolated hypothyroxinemia 3.3%, subclinical hyperthyroidism 3.9%, overt hyperthyroidism 0.5%,
**Liu, Su et al.** 2022	**TSH** 2.5-97.5^th^ pc for SP **FT4** 2.5-97.5^th^ pc for SP	The median level of TSH was 0.85 mU/L	The median level of FT4 was 16.79 pmol/L	Not studied

ATA, anti-thyroid antibodies; DC, dichorionic; GA, gestational age; GW, gestational week; HCG, human chorionic gonadotropin; FT3, free triiodothyronine; FT4, free thyroxine; MC, monochorionic; MoM, multiples of median; MP, multiple pregnancies; pc, percentiles; SP, single pregnancies; TrP, triple pregnancies; TSH, thyroid stimulating hormone; TTTS, twin-to-twin transfusion syndrome; ↑, higher; ↓, lower.

Bolded values refer to studied population (multiple pregnancy and/or twin pregnancy).

### 3.2 TSH levels in TP/MP

TSH examination was performed in 11 out of the studies (25, 30, 31, 33–40). The comparison of TSH levels between SP and TP/MP in the 1^st^ trimester of pregnancy was available in 8 studies. In 5 cases, a lower TSH level in the 1^st^ trimester was found in SP compared to TP (25, 36, 37, 39, 40). Such a phenomenon was not confirmed by Grün et al., Sakaguchi et al. and Ogueh et al., but their studies were characterized by the lowest number of participants among the included papers (33–35). Interestingly, Ogueh et al. showed significantly lower TSH in pregnancies with more than two fetuses compared to SP, but not to TP (35). Regarding the remaining two trimesters, such data were available in articles authored by Grün et al., Sakaguchi et al., Ogueh et al., Dashe et al., and Chen et al. (25, 33–36). In this aspect, Chen et al. reported higher TSH in TP than in SP in the 3^rd^ trimester of pregnancy, while the remaining authors failed to demonstrate statistically significant differences (25). As for other included studies, Hanaoka et al. did not report detailed results for TSH They only mentioned that twin-to-twin transfusion syndrome (TTTS) associated with elevated HCG levels might be the cause of the increase in TH concentrations along with the suppression of TSH (38). At this point, it should be mentioned that Ashoor et al. showed no difference in TSH between TTTS TP and normal TP (37). Rosner et al. and Liu et al. only reported the mean or median values of TSH. However, in our viewpoint, they should not be directly compared due to the variability of the populations and methods used, both laboratory and statistical (30, 31).

### 3.3 TH levels in TP/MP

The results of at least one TH were reported in 10 out of 12 studies, while the comparison of TP/MP and SP was available in 7 studies (25, 30, 31, 33–35, 37, 38, 40, 41). In the 1^st^ trimester, FT4 concentrations were statistically higher in TP than in SP according to Grün et al. (pooled, between 8-15 weeks), Jiang et al. (only between 7-12 GWs, not 4-6 GWs) and Chen et al. (25, 33, 40). Higher levels of TH in TP were not confirmed by Sakaguchi et al. (also for FT3), Ogueh et al., Ashoor et al. and Salek et al. (34, 35, 37, 41). Conversely, Ogueh et al. found higher FT4 concentrations in MP than in SP and TP (35). As for non-comparative studies, Hanaoka et al. reported a gradual decline in TH results after successful TTTS laser treatment, whereas Rosner et al. and Liu et al. provided only direct values as in case of TSH – see **Table 3** (30, 31, 38).

### 3.4 Thyroid disorders in TP/MP

Rosner et al., who studied patients with TP at the beginning of providing health care, reported the prevalence of overt hypothyroidism of 14% while assuming the upper limit of TSH at the level of 2.5 IU/mL and FT4 values proposed by the laboratory as reference (31). Chen et al., who adopted population-adjusted norms established in healthy SP (1^st^ trimester: TSH 0.03-3.6 mU/l, FT4 11.7-19.8 pmol/L), confirmed overt hypothyroidism in 1.8% of cases, while subclinical hypothyroidism in 0.7% of cases. This group was the only one to examine women with TP in this aspect in the 3^rd^ trimester, reporting the prevalence of overt hypothyroidism at the level of 1.3% and subclinical hypothyroidism at 8.2%. Interestingly, the occurrence of subclinical hypothyroidism was lower in TP than in SP in the 1^st^ trimester, while in the 3^rd^ trimester it was higher (25). Šálek et al. reported no cases of overt hypothyroidism in the studied group of 1^st^- trimester TP. However, they claimed a 6.4% prevalence of subclinical hypothyroidism in TP when adopting ranges based on TP population (TSH 0.02-2.95 mU/l, FT4 12.2-23.2 pmol/L). They demonstrated no significant difference between SP and TP. However, all cases of hypothyroidism in TP were subclinical, while 7.6% of SP ones were overt (41).

Chen et al. were the only authors who reported the incidence of isolated hypothyroxinemia and hyperthyroidism in TP. Thus, the percentage of isolated hypothyroxinemia was 4.7% in early TP and 3.3% in late TP, which in both cases it was significantly higher than in SP. Regarding hyperthyroidism, the subclinical form was present in 7.2% in early TP and 3.9% in late TP (more frequently than in SP), and the overt one occurred in 8.5% of early TP, which was more common than in SP. However, in late TP, it was diagnosed in only 0.5% and there was no significant difference compared to SP (25).

### 3.5 Thyroid autoimmunity in TP/MP

**Table 4** presents a summary of 5 studies reporting thyroid autoimmunity in MP (25, 30, 37, 40, 41). The prevalence of autoimmunity reported in TP varied from 10.4% in the study by Liu et al. up to 15.7% in the study by Ashoor et al. (30, 37). Šálek et al. reported TPOAb (+) positivity in 71% of cases of hypothyroid TP compared to 52% in hypothyroid SP with no statistical significance of the difference (41). Ashoor et al. compared DC TP and MC TP regarding the ATA status. However, they found the difference to be in significant (37). This group of authors also reported higher 1^st^ trimester TSH in ATA- positive TP, but FT4 and β-HCG were not different compared to ATA- negative subgroup (37).

**Table 4:** Anti-thyroid antibodies in multiple pregnancies.

Authors and year of publication	Thyroid autoimmunity
**Ashoor, Muto** **et al.** 2013	• The prevalence of TPOAb or TgAb (+) was 15.7% in the whole studied TP population, 14.1% in women in DC TP, 20.7% in MC TP and 10.5% in TTTS group • TSH was higher in ATA (+) group, but FT4 and β-HCG were not different compared to ATA (-) group • TSH was above the 97.5^th^ pc in 29.4% in both ATA (TPOAb and TgAb (+)) groups compared to 2.0% of the ATA (-) group • There was no difference in the proportion of FT4 below the 2.5^th^ pc between the ATA (+) and ATA (-) groups
**Šálek, Dhaifalah et al.** 2019	• The prevalence of TPOAb (+) hypothyroid women was 71% vs 52% for TP and SP, respectively, but the difference was not significant
**Jiang, Sun et al.** 2019	• The prevalence of TPOAb (+) was 14% in women with TP
**Chen, Yang et al.** 2021	The prevalence of TPOAb (+) was 10.8% in women with TP (vs 10.3% in SP – no statistical difference) • During late pregnancy, TSH level did not differ between TPOAb (+) and TPOAb (-) women with both TP and SP
**Liu, Su et al.** 2022	• The prevalence of TPOAb (+) was 10.4% in women with TP

## 4 Discussion

### 4.1 General overview of the results

**Figure 2:**
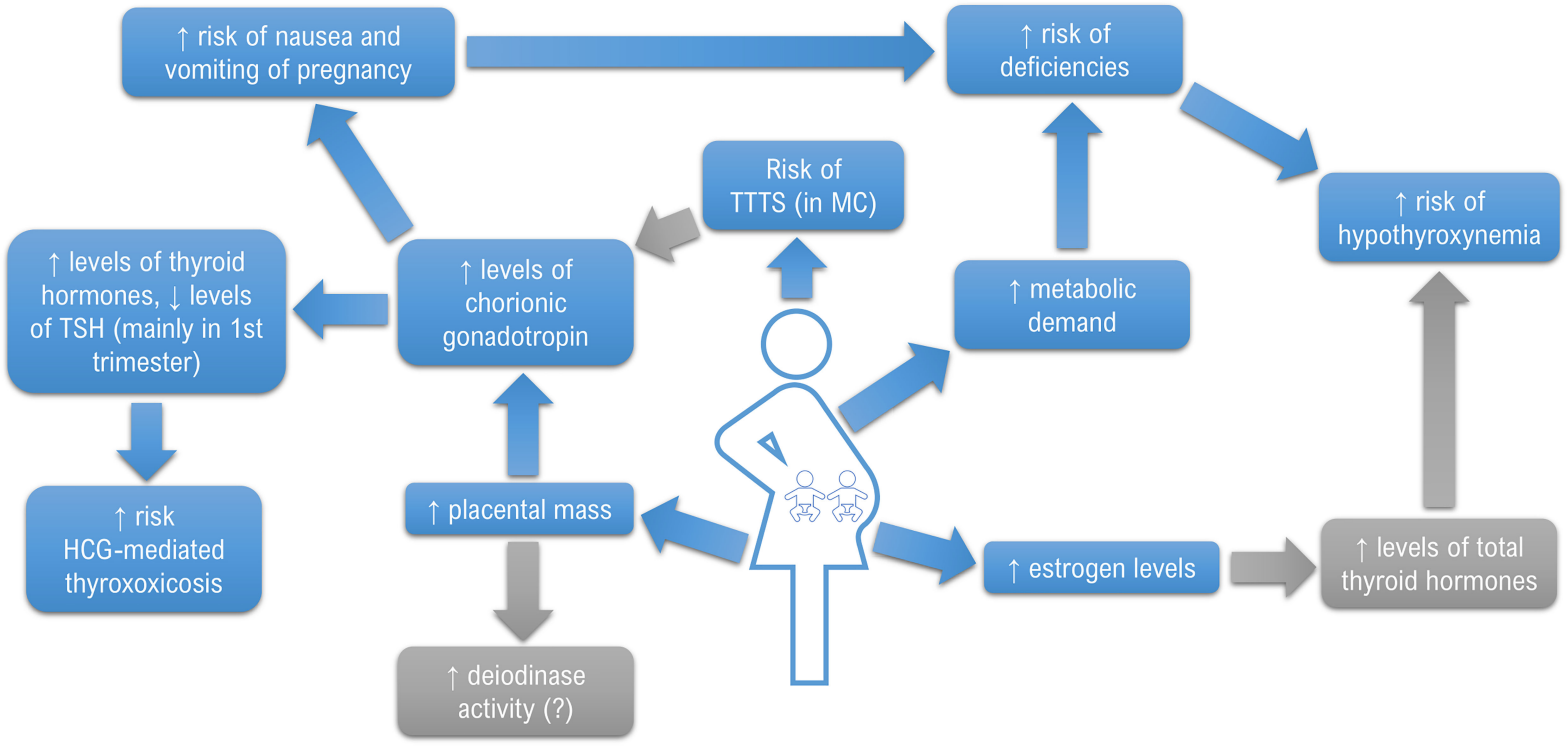
Mechanisms potentially affecting the different thyroid function in multiple pregnancies.

Basing on the data review, the first conclusion that emerges is that there is still too little research-based knowledge concerning the problem. Fortunately, when analyzing the years when the papers were published, it may be concluded that the interest in the field has been growing recently with the increasing occurrence of MP. It is mainly due to the spread of assisted reproductive techniques (ART) and increasing maternal age. According to a study by Monden et al. who based on the data from 2010-2015 obtained from 165 countries, approximately 1.6 million pairs of twins were born annually, about 1 in 40 babies were born as a twin and the direct number of twin deliveries was higher than ever before (44, 45). MPs were found to develop more adverse outcomes than singletons, so it is especially important to act on any modifiable risk factor which could also include the function of the thyroid gland (46, 47). Based on the presented results, it may be concluded that multiple pregnancy may be associated with a decrease in TSH in the 1^st^ trimester and a simultaneous increase in TH concentrations compared to SP, although some studies have failed to prove such differences. Based on a single, yet a large study by Chen et al., it may be concluded that thyroid function disorders are probably more common in women with TP/MP. However, none of the studies showed differences in the frequency of thyroid autoimmunity between SP and TP/MP. The obtained results and postulated mechanisms are summarized in **Figure 2**.

### 4.2 The impact of human chorionic gonadotropin

The analyzed studies were carried out mainly during the 1^st^ trimester of pregnancy, which is probably related to the pathophysiology of MP. Namely, the pattern of HCG secretion is one of the main differences between SP and MP. Half of the studies included in the review focused on this aspect. The results of studies concerning the fluctuations of HCG and its subunit concentrations during pregnancy and its influence on the thyroid axis in MP are presented in **Table 5**.Grün et al., Ogueh et al. and Chen et al. reported a higher 1^st^ trimester peak of HCG in TP/MP compared to SP (25, 33, 35). Conversely, Sakaguchi et al. did not report such a phenomenon. However, similarly to Ogueh et al., they observed that HCG remained higher in the 2^nd^ and 3^rd^ trimester compared to SP as well (34). Grün et al., who performed the measurements at the highest number of timepoints, noted that the increase in the concentration of dimeric HCG was not only higher, but also occurred earlier and lasted longer than in SP (33). Nevertheless, their study did not reveal statistically significant differences between SP and TP as regards HCG levels after the 20^th^ week of gestation (33). Sakaguchi et al. and Ogueh et al. included pregnancies of a greater number of fetuses than twins and reported no differences in HCG levels between TP and multifetal pregnancies (34, 35). Studies in which both HCG and the β-HCG subunits were measured showed that they acted similarly throughout the pregnancy (34, 35). Ashoor et al. reported higher β-HCG in the 1^st^ trimester of TP (37). α-HCG was found to act differently and increase gradually during pregnancy. However, Grün et al. only observed its higher concentration in MP at week 11 (33), whereas Sakaguchi et al. noted it in all trimesters (34).

**Table 5:** Human chorionic gonadotropin, its subunits and its influence on the thyroid function in multiple pregnancies.

Authors and year	Dimer/intact HCG		β-HCG	α-HCG	Correlation with TSH and other TFT
**Grün, Meuris et al.** 1997	• In TP, HCG peak occurred earlier (8^th^ week), was ↑ (mean 171 000 vs 65 500 U/l between 9-11 GWs) and more prolonged than in SP; • In TP, HCG was on average 2.5-fold ↑ than in SP between 8-19 GWs		• In TP, β-HCG peak was ↑ and more prolonged than in SP • β-HCG was ↑ in TP than in SP between 11-19 GWs	• α-HCG image was similar in both TP and SP – progressive and parallel increase with gestational time • α-HCG was significantly ↑ in TP only at 11 GWs	• In both groups the mirror image between HCG and TSH was documented • In TP, the rise in FT4 occurred around 2 weeks after HCG peak • A direct positive correlation between serum HCG and FT4 was observed between 8-15 GWs in TP, but not in SP
	• The ratios of β-HCG/total HCG were similar in SP and TP, and did not vary with gestation time				
**Sakaguchi, Yoshimura et al.** 1998	• In MP, HCG dimers were ↑ than in SP in the 2^nd^ and 3^rd^ trimesters • There were no differences between TP and TrP in HCG levels	• In MP, β-HCG levels were ↑ than in SP in the 2^nd^ and 3^rd^ trimesters		• In MP, α-HCG levels were ↑ than in SP in all trimesters • α-HCG levels rose throughout the pregnancy and did not correlate with HCG dimer or β-HCG in either SP or MP	• No negative correlation between HCG and TSH was found in SP and MP • HCG and β-HCG showed a positive correlation with FT3 and FT4 in SP, but not in MP
	• In both SP and MP, there was a positive correlation between HCG dimer and β-HCG		
**Ogueh, Hawkins et al.** 2000	• HCG was ↑ in MP than in SP in all trimesters		Not studied	Not studied	• Before fetal reduction, there was a positive association between FT4 and HCG and fetal number, and a negative association with TSH • Including all variables, FT4 was related to the number of fetuses (a positive relationship) and TSH (a negative relationship), but not to HCG
**Ashoor, Muto** **et al.** 2013	Not studied		• In the ATA-negative TP with normal outcomes, β-HCG MoM was ↑ than in SP • β-HCG was no different between MC and DC • In the TTTS ATA-negative group, β-HCG was not different compared to normal TP	Not studied	• There was a correlation between log10 TSH MoM and log10 FT4 MoM and log10 free β-HCG MoM, as well as between log10 FT4 MoM and log10 free β-HCG MoM
**Hanaoka, Arata et al.** 2015	• The pre-operative HCG in TTTS was 5.39 MoM HCG in women with SP examined at the same GA in the same institution		Not studied	Not studied	• There was a positive correlation between HCG and FT3 and FT4 in TTTS pre-operatively • When laser therapy was effective, HCG decreased and FT3 and FT4 decreased progressively
**Chen, Yang et al.** 2021	• HCG was ↑ in TP than in SP during early pregnancy		Not studied	Not studied	• In TP, those with subclinical hyperthyroidism during late pregnancy had considerably ↑ HCG in early pregnancy • Women with isolated hypothyroxinemia during early or late pregnancy had ↓ HCG compared to euthyroid women

ATA, anti-thyroid antibodies; DC, dichorionic; GA, gestational age; GW, gestational week; HCG, human chorionic gonadotropin; FT3, free triiodothyronine; FT4, free thyroxine; MC, monochorionic; MoM, multiples of median; MP, multiple pregnancies; SP, single pregnancies; TrP, triple pregnancies; TSH, thyroid stimulating hormone; TTTS, twin-to-twin transfusion syndrome; ↑, higher; ↓, lower.

It is worth noting the actual HCG impact on hormones in the pituitary-thyroid axis. Lockwood et al. found TSH below ≤0.2 mIU/L in 67% of cases of women with HCG >200,000 IU/L and in all cases with HCG >400,000 IU/L. Elevated FT4 was noted in 32% of specimens with HCG concentrations >200,000 IU/L and in 80% with HCG >400,000 IU/L. However, only 6% of women with >200,000 IU/L had the symptoms of hyperthyroidism (48). In this study, 10% of women with HCG >200,000 IU/L were pregnant with twins, and the result of one of them exceeded 400,000 IU/L, but no separate results were reported for this group (48). As regards studies included in the review, Grün et al. discussed a case of transient hyperthyroidism in TP with HCG >200,000 IU/L which confirmed the hypothesis (33). Molar pregnancies with a co-existing live fetus are a remarkable example of the thyrotropic action of extremely high concentrations of HCG in MP. Molar pregnancies were not included in this review due to their different pathophysiology. Such patients face a considerable risk of developing severe hyperthyroidism constituting one of the main challenges for caregivers (49, 50).

According to the majority of studies included in the review, HCG/β-HCG concentrations were characterized by a negative relationship with the level of TSH and a positive relationship with the levels of free TH (33, 35, 37, 38). Sakaguchi et al. were the only ones who found no such correlation (34). Ogueh et al. also studied pregnancies with a higher fetal number than TP. However, after considering all factors, they suggested that the number of fetuses might be a more important factor regulating FT4 or TSH than the concentration of HCG itself, which was also supported by the delayed response of FT4 levels to HCG decrease (35). Grün et al. also demonstrated an approximately 2-week long delay in FT4 response to HCG, while Hanaoka et al. reported a 4-week long delay in TSH response to HCG (33, 38).

Notably, the role of ATA is another aspect in the thyroid response to the stimulating effect of HCG. Korevaar et al. conducted a study in a large group and found that HCG was positively associated with FT4 and negatively associated with TSH in TPOAb-negative women. However, as regards TPOAb-positive women, HCG was not associated with FT4 or TSH (51). Ashoor et al. demonstrated that TSH was higher in ATA-positive group, but FT4 and β-HCG were not different compared to ATA-negative group (37). It is possible that many more factors influence thyroid response to HCG. Based on the results of a study conducted by Koreevar et al. in a group of women with single pregnancies, higher maternal BMI, male fetal sex and maternal parity over 2 were found to be associated with lower thyroid response to HCG stimulation (52).

The authors of available studies indicated that MC TP might differ from DC in terms of HCG/β-HCG concentrations, but data regarding the direction of the differences are inconsistent (53–55). In this context, the aspect raised by Hanaoka et al., i.e., differences in HCG concentrations in TTTS, seems particularly interesting (38). The authors found HCG concentrations in TTTS patients to be higher than previously reported in TP (56, 57). Moreover, after successful laser treatment, HCG, FT3 and FT4 concentrations decreased. The postulated HCG increase mechanism was mostly due to placental hypoxia secondary to impaired blood flow triggered by polyhydramnios in the recipient (54, 58). Lamine et al. described a case of a 30-year-old woman pregnant with MC complicated by TTTS which resulted in severe hyperemesis gravidarum and hyperthyroidism that resolved after successful fetoscopic laser coagulation. In this case, the levels of HCG were markedly increased before and decreased after the procedure with the simultaneous fluctuations of the levels of free TH (58). Conversely, a study by Ashoor et al. did not confirm the association between TTTS and higher levels of free β-HCG or any differences related to chorionicity (37). The above data show that further exploration of differences in HCG secretion depending on chorionicity and TTTS or twin anemia-polycythemia syndrome (TAPS) could reveal interesting data.

### 4.3 Reference ranges

The risk of overtreatment has risen in recent years due to the widespread use of levothyroxine in pregnant women. Sufficient and irrefutable evidence is available to conclude that it is advisable to treat overt hypothyroidism in pregnancy, since it is associated with abnormal neurodevelopment in the offspring, as well as an increased risk of fetal loss, preterm labor and many other pregnancy complications. Standard therapy was confirmed to improve the outcomes (16, 59). As regards the treatment of subclinical hypothyroidism, the matter remains controversial. Firstly, the fact that authors use different definitions of subclinical hypothyroidism in pregnancy constitutes a major problem with the interpretation of individual study results. Moreover, while some studies confirmed a positive impact of treatment on reducing the risk of pregnancy complications such as preterm delivery, a large randomized trial published in 2017 showed that treatment for subclinical hypothyroidism or hypothyroxinemia introduced between 8 and 20 weeks of gestation did not result in significantly better cognitive outcomes in 5- year -old children compared to no treatment (60–62). Similar results were provided by the CATS (Controlled Antenatal Thyroid Screening) and CATS II studies (63–65). However, it was postulated that treatment may have been initiated too late to prevent potential damage in such a case (59). At the same time the actual risk of overtreatment was confirmed by studies showing the so-called U-shaped maternal FT4 relationship with the child’s Intelligence Quotient and child’s gray matter and cortex volume as well as studies associating excessive FT4 with a higher prevalence of attention deficit hyperactivity disorder and behavioral difficulties in offspring (63, 66). Thus, the treatment of subclinical conditions is again becoming more controversial. It is even more difficult as they are only recognized on the basis of laboratory test results. Therefore, in order to diagnose them correctly, reference values must be developed in a reliable and individualized manner (67). Factors to be considered when developing reference ranges for specific populations are described in the American Thyroid Association Guidelines (59). They recommend the inclusion of women without thyroid diseases, with optimal iodine intake and a negative TPOAb status in the determination of reference intervals (59). However, it was also suggested that other factors may also significantly affect TFT, including body mass index, thyroid nodules, or ethnicity. Based on the evidence gathered, the authors of the present review suggest that the number of fetuses should also be taken into account. It is debatable whether different reference values should be used for women with MC than for women with DC TP. Previously, a hypothesis was proposed that women who became spontaneously pregnant with DC might have a different pituitary function from those with current or past singleton pregnancies (68). They were characterized by higher levels of gonadotropins, FSH in particular. They were also statistically taller, with earlier menarche and earlier menopause, which might also be related to a globally differently functioning pituitary (69–72). At present, these populations certainly differ in the frequency of conception through ART, although it was postulated that ART might have an impact on MC twinning, especially blastocyst transfer (73). Little reference was made to chorionicity in the papers included in the review. Ashoor et al. found no difference in the values of β-HCG, TSH and FT4 and ATA prevalence between DC and MC, but the population did not differ in terms of the frequency of assisted conception (51.3% DC and 48.3% MC) in their study (37). Rosner et al., who studied patients starting maternity care, reported no difference in overt hypothyroidism rates between MC and DC (31).

Our review showed that four groups of authors attempted at creating TP-specific reference ranges (36, 37, 39–41). The direct comparison of values is of limited importance here as the authors used different determination methodologies (**Supplementary Material 1**). However, it is worth mentioning that the lower limit of TSH near the end of the 1^st^ trimester of TP was accounted for almost zero in all those cases, i.e., on the verge of the sensitivity of the method (36, 37, 39–41). According to Ashoor et al., the lowest upper limit of TSH was 2.35 mIU/L for Caucasian women with TP and 1.82 mIU/L for African women with TP, both in groups with BMIs <25 during the 11^th^ week of TP (37). Interestingly, Jiang et al. who examined patients twice in the first 12 weeks of gestation noted significantly lower TSH values at 7-12 weeks compared to 4-6 weeks and suggested the potential need for separate references at that crucial time (40). Ashoor et al. provided separate reference ranges depending on the age, BMI and ethnicity of TP patients, which resulted in noticeable differences in values (37). Similar observations on the population of women with single pregnancies were also published by Laurberg et al. (74).

### 4.4 Future directions

The problem has not been fully elucidated so far. Firstly, subsequent studies should be conducted to establish population-based reference ranges for each trimester for women with MP, including TP in the first place as the most common type. In our opinion, since individual countries differ in basic demographic parameters, ethnicity, iodine supply, fortification of food, thyroid disease profile and the frequency of autoimmune diseases, the reference ranges should at least be created at the national level. Nevertheless, such studies should also focus on the influence of thyroid function in MP on the final pregnancy outcomes, for example by assessing the frequency of maternal and fetal complications or children’s intellectual achievements. The difficulty in establishing such reference ranges is due to the fact that multiple pregnancy is by definition a pathological state. Normally, physiological mechanisms, such as the stimulation of the thyroid gland by HCG, may take the form of a pathology, given the excessive levels of this thyrotropic hormone. It would also be especially valuable to note the type of conception (ART or spontaneous) and chorionicity in studies assessing thyroid function in TP. Research should include information on the supply of iodine in a given population in the studied years and on the applied population-based measures to prevent deficiencies, e.g., salt fortification. Data should also be provided on autoimmunity, i.e., TPOAb, TgAb and TSHR antibodies. It would also be helpful to investigate whether the sera of women with twin pregnancies were more likely to produce laboratory errors, for example by cross-reacting with HCG.

Moreover, it would be valuable to establish reference parameters for fetal thyroid dimensions in TP as in case of SP, as well as to compare the results of children born from MP. According to some authors, congenital hypothyroidism was relatively common in children born from multiple pregnancies (75, 76). It would be interesting to explore potential general hormonal differences between women who conceive twins spontaneously and others in the context of the hypothalamic-pituitary-thyroid axis.

### 4.5 Advantages and limitations

The advantage of the review is the novelty of the undertaken subject. To our knowledge, no similar systematic review has been conducted so far. The study discusses the theory, the results of available research and indicates future directions. However, it is not free from limitations. One of the main limitations is associated with the lack of the statistical synthesis of research results or meta-analysis. Nevertheless, in the opinion of the present authors, performing it in a reliable manner would be impossible. Firstly, the studies included in the review were carried out in different populations. Secondly, various laboratory methods were used, which is of great importance for the results obtained. Another limitation is related to the fact that the included studies lacked potentially relevant data. The authors rarely reported maternal age, BMI, parity or chorionicity. Moreover, the vast majority of studies only considered TP, so the results could not be extrapolated to other types of MP. None of the authors reported if antibodies against TSH receptors were tested, although they are the main factor differentiating gestational thyrotoxicosis from Graves’ disease. However, we do not expect that it is associated with high bias due to the rarity of pregnancy-onset disease (77). Therefore, the quality of the included studies is another limitation (see details in **Supplementary Material 2**). When evaluated with the AXIS tool, the lowest score achieved was 10 out of 20, while the highest was 16 out of 20, with the average of 14.5 ± 1.8. Noticeably more points were scored in newer papers, one reason for that being the possible gradual implementation of good clinical practice and appropriate reporting guidelines over time. The biggest problem was associated with the failure to report the phenomenon of non-respondents and the lack of the analysis of the group size as well as the strength of statistical tests. Regrettably, considering the prevalence of the phenomenon in the general population, one may have concerns about the sufficient power of the tests performed in studies involving few or over a dozen so patients. Furthermore, a substantial number of authors did not discuss the limitations of their own manuscripts. Furthermore, some of them focused on even more selected groups than MP, e.g., only MP conceived as a result of ART or only TP suffering from TTTS, which also certainly limits their representativeness for all MPs.

## 5 Conclusions

Women with MP seem to have a different thyroid function than women with SP. Currently, little research-based knowledge is available on the topic, but it seems that MPs are most likely characterized by higher HCG levels, which has an impact on TSH and free TH levels. Such differences are mainly expressed in the 1^st^ trimester of pregnancy. Separate population-based reference ranges are needed to diagnose individual thyroid diseases in MP correctly and to avoid unnecessary treatment. More research is needed to fulfill the underlying knowledge gaps.

## Data availability statement

The original contributions presented in the study are included in the article/**Supplementary Material**. Further inquiries can be directed to the corresponding author.

## Author contributions

Conceptualization: MZ, KK-K. Methodology: MZ, MC. Investigation: MZ, MO, KK-K, IS. Resources: MZ, MO, IS. Abstracts screening: MZ, MC. Full-text screening: IS, KK-K. Data collection: MZ, MO. Data curation: MZ, MO, MC, KK-K. Risk of bias assessment: MZ. Writing—original draft preparation: MZ, KK-K, MO, MC. Writing—review and editing: KK-K, IS, MC. Supervision: KK-K, IS, MC. Project administration: MZ. Funding acquisition, KK-K, MC. All authors contributed to the article and approved the submitted version.
