# Supplementary material for: Maternal thyroid function in multiple pregnancies – a systematic review
Source: Front Endocrinol (Lausanne). 2023 Jan 17;13:1044655. doi: 10.3389/fendo.2022.1044655 (PMC9887021; doi:10.3389/fendo.2022.1044655)
Supplement: Supplementary file 1 [file Table_1.docx]

Supplementary Material 1. Methodology of laboratory examinations conducted in individual studies.

| **Authors, year and country of publication** | **Studied parameters** | **Parameter** | **Method** | **Analyzer** |
| --- | --- | --- | --- | --- |
| **Grün, Meuris et al.**  1997  **Belgium** | hCG, α-hCG,  β-hCG, TSH, FT4 | FT4 | RIA | Clinical Assays, Baxter, Cambridge, MA, USA |
|  |  | TSH | RIA | Abbott Laboratories, Chicago, IL, USA |
|  |  | hCG, α-hCG,  β-hCG | IRMA | BioMe ́rieux, Marcy-l’Etoile, FR |
| **Sakaguchi, Yoshimura et al.**  1998  **Japan** | hCG, α-hCG,  β-hCG, TSH, FT3,  FT4, TSA | hCG | DELFIA | Pharmacia Co.,Tokyo, JP |
|  |  | α-hCG | RIA | Mitsubishi Chemical B.C.L.Co.,Tokyo, JP |
|  |  | β-hCG | IRMA | Ballelza; CIS Co.,Tokyo, JP |
|  |  | FT4, FT3 | RIA | Amerlex-MAB; Codack Japan Co., Tokyo, JP |
|  |  | TSH | IRMA | Spack-S; Daiichi Radioisotope Laboratories, Tokyo, JP |
| **Ogueh, Hawkins et al.**  2000  **United Kingdom** | TSH, FT4, hCG | TSH, FT4 | ECLIA | Elecsys; Roche Diagnostic Ltd, Lewes, UK |
|  |  | hCG | FIA | Pharmacia Wallac, Milton Keynes, Bucks, UK |
| **Dashe, Casey  et al.**  2005  **United States of America** | TSH | TSH | CLIA | Immulite 2000; Diagnostic Products Corporation, Los Angeles, CA, USA |
| **Ashoor, Muto  et al.**  2013  **United Kingdom** | β-hCG, TSH, FT4, TPOAb, TgAb | β-hCG | DELFIA | DELFIA Xpress; PerkinElmer, Waltham, MA, USA |
|  |  | TSH, FT4, TPOAb, TgAb | CLIA | Advia Centaur; Siemens Healthcare Diagnostics, Surrey, UK |
| **Hanaoka, Arata et al.**  2015  **Japan** | hCG, TSH, FT3, FT4 | hCG, TSH, FT3, FT4 | CLIA | Immulite; Diagnostic Products Corporation, Los Angeles, CA, USA |
| **Rosner, Fox et al.**  2017  **United States of America** | TSH, FT4 | hCG, TSH, FT3, FT4 | Not reported | |
| **Šálek, Dhaifalah et al.**  2018  **Czech Republic** | TSH, FT4, TPOAb | TSH, FT4, TPOAb | CLIA | ARCHOTECT ci16200; Abbott Laboratories, Chicago, IL, USA |
| **Šálek, Dhaifalah et al.**  2019  **Czech Republic** | TSH, FT4, TPOAb | TSH, FT4, TPOAb | CLIA | ARCHOTECT ci16200; Abbott Laboratories, Chicago, IL, USA |
| **Jiang, Sun et al.**  2019  **China** | TSH, FT4, TPOAb | TSH, FT4 | ECLIA | Advia Centaur; Siemens Healthcare Diagnostics, Tarrytown, NY, USA |
|  |  | TPOAb | ECLIA | Cobas 601; Roche Diagnostics, Mannheim, DE |
| **Chen, Yang et al.**  2021  **China** | hCG, TSH, FT4, TPOAb | TSH, FT4, TPOAb | CLIA | ARCHITECT i2000; Abbott Laboratories, Chicago, IL, USA |
|  |  | hCG | CLIA | Immulite 2000 XPi; Siemens Healthcare Diagnostics, Deerfield, IL, USA |
| **Liu, Su et al.**  2022  **China** | TSH, FT4, TPOAb | TSH, FT4, TPOAb | FIA, CLIA | Advia Centaur; Siemens Healthcare Diagnostics, Munich, DE |

CLIA – chemiluminescence immunoassay; DELFIA - dissociation-enhanced lanthanide fluorescence immunoassay; ECLIA – electrochemiluminescence immunoassay; FIA – fluoroimmunoassay; IRMA – immunoradimetric assay; RIA – radioimmunoassay
