# Supplementary material for: Maternal thyroid function in multiple pregnancies – a systematic review
Source: Front Endocrinol (Lausanne). 2023 Jan 17;13:1044655. doi: 10.3389/fendo.2022.1044655 (PMC9887021; doi:10.3389/fendo.2022.1044655)
Supplement: Supplementary file 2 [file Table_2.docx]

Supplementary Material 2. Risk of bias assessment.

Note: This is based on AXIS tool developed for a risk of bias assessment (Downes, Brennan et al. 2016)

Possible answers: Yes / No / Do not know / Not reported (NR) / Comment

The assessment refers to the population of women with multiple pregnancies included in each study.

|  | (Grün, Meuris et al. 1997) | (Sakaguchi, Yoshimura et al. 1998) | (Ogueh, Hawkins et al. 2000) | (Dashe, Casey et al. 2005) | (Ashoor, Muto et al. 2013) | (Hanaoka, Arata et al. 2015) | (Rosner, Fox et al. 2017) | (Šálek, Dhaifalah et al. 2018) | (Salek, Dhaifalah et al. 2019) | (Jiang, Sun et al. 2019) | (Chen, Yang et al. 2021) | (Liu, Su et al. 2022) |
| --- | --- | --- | --- | --- | --- | --- | --- | --- | --- | --- | --- | --- |
| **Introduction** | | | | | | | | | | | | |
| 1. Were the aims/objectives of the study clear? | Yes | Yes | Yes | Yes | Yes | Yes | Yes | Yes | Yes | Yes | Yes | Yes |
| **Methods** | | | | | | | | | | | | |
| 2 Was the study design appropriate for the stated aim(s)? | Yes | Yes | Yes | Yes | Yes | Yes | Yes | Yes | Yes | Yes | Yes | Yes |
| 3 Was the sample size justified? | No | No | No | No | No | No | No | No | No | No | No | No |
| 4 Was the target/reference population clearly defined? (Is it clear who the research was about?) | Yes | Yes | Yes | Yes | Yes | Yes | Yes | Yes | Yes | Yes | Yes | Yes |
| 5 Was the sample frame taken from an appropriate population base so that it closely represented the target/reference population under investigation? | No^1^ | NR | Yes | Yes | Yes | No^2^ | Yes | Yes | Yes | Yes | Yes | Yes |
| 6 Was the selection process likely to select subjects/participants that were representative of the target/reference population under investigation? | No^1^ | NR | No^1^ | Yes | Yes | No^2^ | Yes | Yes | Yes | Yes | Yes | Yes |
| 7 Were measures undertaken to address and categorize non-responders? | No | No | No | No | No | No | No | No | No | No | No | No |
| 8 Were the risk factor and outcome variables measured appropriate to the aims of the study? | Yes | Yes | Yes | Yes | Yes | Yes | Yes | Yes | Yes | Yes | Yes | Yes |
| 9 Were the risk factor and outcome variables measured correctly using instruments/ measurements that had been trialled, piloted or published previously? | Yes | Yes | Yes | Yes | Yes | Yes | Yes | Yes | Yes | Yes | Yes | Yes |
| 10 Is it clear what was used to determined statistical significance and/or precision estimates? (e.g., p values, CIs) | Yes | Yes | Yes | Yes | Yes | Yes | Yes | Yes | Yes | Yes | Yes | Yes |
| 11 Were the methods (including statistical methods) sufficiently described to enable them to be repeated? | Yes | No | Yes | Yes | Yes | Yes | Partially^3^ | Yes | Yes | Yes | Yes | Yes |
| **Results** | | | | | | | | | | | | |
| 12 Were the basic data adequately described? | Yes | Yes | Yes | Yes | Yes | Yes | Yes | Yes | Yes | Yes | Yes | Yes |
| 13 Does the response rate raise concerns about non-response bias? | NR | NR | NR | NR | NR | NR | NR | NR | NR | NR | NR | NR |
| 14 If appropriate, was information about non-responders described? | No | No | No | No | No | No | No | No | No | No | No | No |
| 15 Were the results internally consistent? | Yes | Yes | Yes | Yes | Yes | Yes | Yes | Yes | Yes | Yes | Yes | Yes |
| 16 Were the results for the analyses described in the methods, presented? | Yes | Yes | Yes | Yes | Yes | Yes | Yes | Yes | Yes | Yes | Yes | Yes |
| **Discussion** | | | | | | | | | | | | |
| 17 Were the authors’ discussions and conclusions justified by the results? | Yes | Yes | Yes | Yes | Yes | Yes | Yes | Yes | Yes | Yes | Yes | Yes |
| 18 Were the limitations of the study discussed? | No | No | Yes | No | No | Yes | Yes | Yes | Yes | Yes | Yes | Yes |
| **Others** | | | | | | | | | | | | |
| 19 Were there any funding sources or conflicts of interest that may affect the authors’ interpretation of the results? | No | NR | NR | NR | No | No | No | NR | No | No | No | No |
| 20 Was ethical approval or consent of participants attained? | Yes | NR | Yes | Yes | Yes | Yes | Yes | Yes | Yes | Yes | Yes | Yes |
| Total Score | 13/20 | 10/20 | 14/20 | 14/20 | 15/20 | 14/20 | 15-16/20 | 15/20 | 16/20 | 16/20 | 16/20 | 16/20 |

^1^ Whole study group underwent various assisted reproductive techniques what can be the source of some bias

^2^Study group consisted of women with twin-to-twin transfusion syndrome what can be the source of some bias

^3^Details on laboratory methods were not provided

Ashoor, G., O. Muto, L. C. Poon, M. Muhaisen and K. H. Nicolaides (2013). "Maternal thyroid function at gestational weeks 11-13 in twin pregnancies." Thyroid **23**(9): 1165-1171.

Chen, Z., X. Yang, C. Zhang, Z. Ding, Y. Zhang, T. I. M. Korevaar and J. Fan (2021). "Thyroid Function Test Abnormalities in Twin Pregnancies." Thyroid **31**(4): 572-579.

Dashe, J. S., B. M. Casey, C. E. Wells, D. D. McIntire, E. W. Byrd, K. J. Leveno and F. G. Cunningham (2005). "Thyroid-stimulating hormone in singleton and twin pregnancy: importance of gestational age-specific reference ranges." Obstet Gynecol **106**(4): 753-757.

Downes, M. J., M. L. Brennan, H. C. Williams and R. S. Dean (2016). "Development of a critical appraisal tool to assess the quality of cross-sectional studies (AXIS)." BMJ Open **6**(12): e011458.

Grün, J. P., S. Meuris, P. De Nayer and D. Glinoer (1997). "The thyrotrophic role of human chorionic gonadotrophin (hCG) in the early stages of twin (versus single) pregnancies." Clin Endocrinol (Oxf) **46**(6): 719-725.

Hanaoka, M., N. Arata and H. Sago (2015). "Change of maternal thyroid function in twin-twin transfusion syndrome." Endocr J **62**(10): 949-952.

Jiang, Y. X., W. J. Sun, Y. Zhang, Y. Huang, Y. Y. Huang, G. Z. Lu, J. Q. Zhang, Y. Gao, H. X. Yang and X. H. Guo (2019). "Thyroid function of twin-pregnant women in early pregnancy." Chin Med J (Engl) **132**(17): 2033-2038.

Liu, X. S., X. J. Su, G. H. Li, S. J. Huang, Y. Liu, H. X. Sun and Q. L. Du (2022). "Maternal Thyroid Function and Birth Weight in Twins." Endocrinology **163**(8).

Ogueh, O., A. P. Hawkins, A. Abbas, G. D. Carter, K. H. Nicolaides and M. R. Johnson (2000). "Maternal thyroid function in multifetal pregnancies before and after fetal reduction." J Endocrinol **164**(1): 7-11.

Rosner, J. Y., N. S. Fox, D. Saltzman, A. Rebarber and S. Gupta (2017). "The Effect of Treated Overt Hypothyroidism on Outcomes in Twin Pregnancies." American Journal of Perinatology **34**(14): 1447-1450.

Sakaguchi, N., M. Yoshimura, M. Nishikawa, N. Yoshikawa, N. Toyoda, T. Yonemoto, Y. Ogawa, S. Tabata, T. Tokoro, S. Fukunaga, K. Sugano, H. Kanzaki and M. Inada (1998). "Maternal thyroid function in multiple pregnancy: the variable thyrotropic activity of human chorionic gonadotropin." Horm Metab Res **30**(11): 689-693.

Salek, T., I. Dhaifalah, D. Langova and J. Havalova (2019). "The prevalence of maternal hypothyroidism in first trimester screening from 11 to 14 weeks of gestation." Biomed Pap Med Fac Univ Palacky Olomouc Czech Repub **163**(3): 265-268.

Šálek, T., I. Dhaifalah, D. Langova and J. Havalová (2018). "Maternal thyroid-stimulating hormone reference ranges for first trimester screening from 11 to 14 weeks of gestation." J Clin Lab Anal **32**(6): e22405.
